# Differential expression of *IDA* (*INFLORESCENCE DEFICIENT IN ABSCISSION*)-like genes in *Nicotiana benthamiana* during corolla abscission, stem growth and water stress

**DOI:** 10.1186/s12870-020-2250-8

**Published:** 2020-01-20

**Authors:** Daniel Ventimilla, Concha Domingo, Daniel González-Ibeas, Manuel Talon, Francisco R. Tadeo

**Affiliations:** Centro de Genómica, Instituto Valenciano de Investigaciones Agrarias (IVIA), CV-315, Km 10,7 Moncada, E-46113 Valencia, Spain

**Keywords:** Abiotic stress, Cell separation, Cis-acting regulatory elements, Gene expression, LRR-RLKs, Phylogeny, Signaling peptide, Solanaceae, Vegetative growth

## Abstract

**Background:**

IDA (INFLORESCENCE DEFICIENT IN ABSCISSION)-like signaling peptides and the associated HAE (HAESA)-like family of receptor kinases were originally reported in the model plant *Arabidopsis thaliana* (Arabidopsis) to be deeply involved in the regulation of abscission. Actually, IDA peptides, as cell-to-cell communication elements, appear to be implicated in many developmental processes that rely on cell separation events, and even in the responses to abiotic stresses. However, the knowledge related to the molecular machinery regulating abscission in economically important crops is scarce. In this work, we determined the conservation and phylogeny of the *IDA*-like and *HAE*-like gene families in relevant species of the Solanaceae family and analyzed the expression of these genes in the allopolyploid *Nicotiana benthamiana,* in order to identify members involved in abscission, stem growth and in the response to drought conditions.

**Results:**

The phylogenetic relationships among the *IDA*-like members of the Solanaceae studied, grouped the two pairs of NbenIDA1 and NbenIDA2 protein homeologs with the Arabidopsis prepropeptides related to abscission. Analysis of promoter regions searching for regulatory elements showed that these two pairs of homeologs contained both hormonal and drought response elements, although *NbenIDA2A* lacked the hormonal regulatory elements. Expression analyses showed that the pair of *NbenIDA1* homeologs were upregulated during corolla abscission. *NbenIDA1* and *NbenIDA2* pairs showed tissue differential expression under water stress conditions, since *NbenIDA1* homeologs were highly expressed in stressed leaves while *NbenIDA2* homeologs, especially *NbenIDA2B*, were highly expressed in stressed roots. In non-stressed active growing plants, nodes and internodes were the tissues with the highest expression levels of all members of the *IDA*-like family and their putative *HAE*-like receptors.

**Conclusion:**

Our results suggest that the pair of *NbenIDA1* homeologs are involved in the natural process of corolla abscission while both pairs of *NbenIDA1* and *NbenIDA2* homeologs are implicated in the response to water stress. The data also suggest that IDA peptides may be important during stem growth and development. These results provide additional evidence that the functional module formed by IDA peptides and its receptor kinases, as defined in Arabidopsis, may also be conserved in Solanaceae.

## Background

The significance of the *INFLORESCE DEFICIENT IN ABSCISSION* (*IDA*)-like gene family is primary associated with the observation that *AtIDA* was deeply involved in the regulation of the abscission of floral organs and cauline leaves in *Arabidopsis thaliana* (Arabidopsis) [1–3]. Abscission is an active, organized and highly coordinated cell separation process allowing the detachment of entire vegetative and reproductive organs through the modification of cell-to-cell adhesion and breakdown of cell walls at specific sites on the plant body known as abscission zones (AZs), a discrete group of functionally specialized cells (for a review, see [4]). From an evolutionary point of view, abscission is a very favorable process that has several advantages such as seed dispersal as well as the shedding of no longer needed, damaged or infected organs. In addition, the abscission process is related to other processes such as senescence, pathogen defense and drought stress tolerance [5]. Abscission of aerial organs, on the other hand, may become a major limiting factor of yield in an agricultural context. It is widely accepted that the control of abscission in Arabidopsis requires physical interaction of the hormonal peptide AtIDA, a pair of redundant receptor-like protein kinases, HAESA (HAE) and HAESA- LIKE2 (HSL2), and SOMATIC EMBRYOGENESIS RECEPTOR-LIKE KINASE (SERK) co-receptors (for a review, see [6]). This interaction activates a signal transduction through a downstream MAP kinase cascade that leads to the expression of multiple abscission-related hydrolytic enzymes such as pectin-methylesterases, polygalacturonases, cellulases, xyloglucan-endotransglycosylase/hydrolases and expansins [7, 8]. The release of this set of enzymes cause the disassembly of the cell wall and the dissolution of the middle lamella, resulting in the detachment of floral organs in Arabidopsis. Furthermore, in Arabidopsis *ida* mutants, petals remain indefinitely attached to the flower [1]. It has been also shown that synthetic IDA peptides were able to induce early floral abscission in Arabidopsis flowers [9].

The function of IDA peptides, as cell-to-cell communication elements, does not appear to be solely restricted to their roles in abscission. The activity of these peptides has also recently been involved in other developmental processes in Arabidopsis all of them settled on cell separation events such as the emergence of lateral roots and the root cap sloughing [10, 11]. In addition, IDA peptides have recently been involved in the response to abiotic stresses. Expression analysis of Arabidopsis *AtIDL6* and *AtIDL7*, for instance, revealed that these two genes are rapidly induced during various stress treatments [12]. It was subsequently determined experimentally that these peptides were involved in the stress response as modulators of reactive oxygen species (ROS) signaling [13]. Furthermore, treatments with AtIDL6 and AtIDL7 peptides caused downregulation of important stress-response key regulators like ZINC FINGER PROTEIN and WRKY transcription factors.

In addition to Arabidopsis, *IDA*-like genes have been identified in some crop species associated with organ abscission and the emergence of lateral roots [14] as well. Regarding organ abscission, particular members of the *IDA*-like gene family of tomato (*SlIDA1*), soybean (*GmIDA2a*), citrus (*CitIDA3*), litchi (*LcIDL1*), oil palm (*EgIDA5*) and yellow lupine (*LlIDA*) were highly expressed in leaf, flower or fruit abscission zones during abscission [15–19]. In addition, the *AtIDA* homologues of citrus (*CitIDA3*) and litchi (*LcIDA1*) were able to induce earlier floral organ abscission and to rescue the *ida2* abscission deficiency when ectopically expressed in Arabidopsis [16, 20]. All these data, together with the positive affect of treatments with synthetic IDA peptides on triggering organ abscission [9, 19, 21] strongly suggest conserved functions of *IDA*-like genes in regulating cell separation events during organ abscission.

Solanaceae is a large plant family with approximately 90 genera comprising more than 3000 species found on almost all continents. Solanaceae is also one of the most economically important families worldwide. Some species of this family such as tomato (*Solanum lycopersicum*), potato (*S. tuberosum*), eggplant (aubergine; *S. melongena*) and pepper (*Capsicum annum*) are of great relevance as a human food source. Overall, more than 29 million hectares of these Solanaceae food species were cultivated globally in 2016, producing 644 million metric tons with a net production value of more than 146 billion US dollars (http://www.fao.org/faostat). Thus, in addition to being important in human nutrition, they are also relevant in economic and social terms. Other Solanaceae such as tobacco (*Nicotiana* spp.) have medical importance as a source of plant drugs while *Nicotiana benthamiana* is considered a relevant model organism for the study of plant-microbe interactions and also in plant molecular research and biotechnology [22, 23]. In this work, firstly, we determined the conservation and phylogeny of the *IDA*-like and *HAE*-like gene families by taking advantage of the free availability of the diploid genome sequences of tomato, eggplant, pepper, *N. sylvestris*, *N. tomentosiformis,* the allopolyploids *N. tabacum* and *N. benthamiana*, and the double haploid genome sequence of potato in the Solanaceae Genomic Network (SGN; https://solgenomics.net/). Allopolyploidy is a type of whole genome duplication derived from hybridization of two or more diverged taxa, that primarily occurs through the fusion of unreduced (2*n*) gametes. The result of this kind of genome merging is the occurrence of pairs of homologous genes from each of the diploid parents in the allopolyploid genome, called homeologs [24]. Therefore, in the allopolyploid genomes of *N. tabacum* and *N. benthamiana* we will likely find pairs of homeologs for many of the members of the gene families.

In order to identify and discriminate members involved in organ abscission, stem growth and in the response to drought conditions, we examined the expression of the homeolog genes of the *N. benthamiana IDA*-like and *HAE*-like families. The abscission of the corolla, the only organ that undergoes abscission in *N. benthamiana,* should be highly similar to that reported in *N. tabacum* [25]. The detachment of the corolla is due to the dissolution of the middle lamella and apparently to the disintegration of the parenchymal cells in its basal zone, a process that results in the detachment of the senescent corolla. Furthermore, the effect of water stress on the species of this contrasted genus has been the subject of major research and the physiological responses of these plants are also well known [26–29].

## Results

### The *IDA*-like gene family in the Solanaceae

Table 1 summarizes all *IDA*-like genes identified in our search in representative species of the genus *Nicotiana* such as *N. sylvestris*, *N. tomentosiformis*, *N. tabacum* and *N. benthamiana* in addition to other Solanaceae of agronomic interest such as tomato, potato, eggplant and pepper. All prepropeptides identified share two relevant characteristics, a signal peptide targeting the protein to the apoplast through the secretory pathway and a highly conserved C-terminal signature termed PIP motif typical of this gene family [1].

**Table 1:** IDA-like gene families in species of the Solanaceae family (genome localization from different Sol Genomics Network databases [30]). All prepropeptides are predicted to be localized in the secretory pathway according to TargetP [31] and SignalP-5.0 [32]

Gene name	Genome localization	Prepropeptide length (aa)	Predicted signal peptide length (aa)	PIP domain
NsylIDA1	Nsyl_KD945166.1:74265..74582 forward	105	39	PIPPSAPSKRHN
NsylIDA2	Nsyl_KD978144.1:88678..88980 reverse	100	32	PIPPSAPSKRHN
NsylIDA3	Nsyl_KD951180.1:40337..40579 forward	80	32	PIPPSAPSKRHN
NsylIDA4	Nsyl_KD977536.1:13349..13576 forward	75	22	PIPPSAPSQRHN
NsylIDA5	Nsyl_KD962079.1:38313..38564 forward	83	30	PIPASGPSRKHN
NtomIDA1	Ntom_KB972926.1:26032..26325 forward	97	37	PIPPSAPSKRHN
NtomIDA2	Ntom_KB954314.1:53025..53614 forward	96	32	PIPPSAPSKRHN
NtomIDA3	Ntom_KB969023.1:33965..34204 forward	79	31	PIPPSAPSKRHN
NtomIDA4	Ntom_KB956501.1:19193..19405 reverse	70	22	PIPPSAPSQRHN
NtomIDA5	Ntom_KB958630.1:30910..31161 reverse	83	30	PIPASGPSRKHN
NtabIDA1A	Ntab-BX_AWOK-SS18147:707412..707735 reverse	107	48	PIPPSAPSKRHN
NtabIDA1B	Ntab-BX_AWOK-SS9960:271769..272062 forward	97	37	PIPPSAPSKRHN
NtabIDA2A	Ntab-BX_AWOK-SS12153:24919..25221 reverse	100	32	PIPPSAPSKRHN
NtabIDA2B	Ntab-BX_AWOK-SS20685:67080..68370 reverse	96	32	PIPPSAPSKRHN
NtabIDA3A	Ntab-BX_AWOK-SS473:166199..166441 reverse	80	32	PIPPSAPSKRHN
NtabIDA3B	Ntab-BX_AWOK-SS2799:946688..946927 forward	79	31	PIPPSAPSKRHN
NtabIDA4A	Ntab-BX_AWOK-SS18001:26098..26325 forward	75	22	PIPPSAPSQRHN
NtabIDA4B	Ntab-BX_AWOK-SS12176:491822..492033 reverse	70	22	PIPPSAPSQRHN
NtabIDA5A	Ntab-BX_AWOK-SS18104:315608..315859 reverse	83	30	PIPASGPSRKHN
NtabIDA5B	Ntab-BX_AWOK-SS9524:125323..125574 forward	83	30	PIPASGPSRKHN
NbenIDA1A	Niben101Scf00570:62104..62373 reverse	90	36	PIPPSAPSK-----
NbenIDA1B	Niben101Scf01338:640730..641035 forward	101	35	PIPPSAPSKRHN
NbenIDA2A	Niben101Scf23219:7370..7663 reverse	97	32	PIPPSAPSKRHN
NbenIDA2B	Niben101Scf03368:114599..114892 reverse	97	32	PIPPSAPSKRHN
NbenIDA3A	Niben101Scf18667:206436..206678 forward	80	32	PIPPSAPSKRHN
NbenIDA3B	Niben101Scf01180:267334..267576 reverse	80	32	PIPPSAPSKRHN
NbenIDA4	Niben101Scf19133:87532..87771 forward	79	25	PIPPSAPSQRHN
NbenIDA5A	Niben101Scf03848:699324..699575 forward	83	30	PIPASGPSRKHN
NbenIDA5B	Niben101Scf02135:404883..405122 reverse	79	26	PIPASGPSRKHN
SlycIDA1	SL3.0ch05:4200134..4200439 forward	101	36	PIPPSAPSKRHN
SlycIDA2	SL3.0ch06:38623220..38623453 forward	77	30	PIPPSAPSKRHN
SlycIDA3	SL3.0ch04:5799910..5800149 forward	79	27	PIPPSSPSKRHN
SlycIDA4	SL3.0ch07:58068277..58068558 reverse	93	34	PIPPSAPSKRCN
SlycIDA5	SL3.0ch05:1629558..1629893 forward	111	29	LIPPSGPSRRHN
SlycIDA6	SL3.0ch09:540104..540379 reverse	91	26	PIPPSAPSCRSS
SlycIDA7	SL3.0ch09:546577..546855 reverse	92	27	PLPPSAPSCRSS
SlycIDA8	SL3.0ch11:533813..534061 reverse	82	28	PIPASGPSRKHN
StubIDA1	PGSC0003DMB000000071:252349..252627 reverse	92	27	PIPPSAPSCRSS
StubIDA2	PGSC0003DMB000000131:879373..879621 forward	82	28	PIPASGPSRKHN
StubIDA3	PGSC0003DMB000000243:905236..905523 forward	95	29	PVPPSGPSRRHN
StubIDA4	PGSC0003DMB000000410:16621..16935 reverse	104	36	PIPPSAPSKRHN
StubIDA5	PGSC0003DMB000000420:159814..160050 forward	78	26	PIPPSSPSKRHN
StubIDA6	PGSC0003DMB000000461:377302..377535 forward	77	30	PIPPSAPSKRHN
StubIDA7	PGSC0003DMB000000592:149451..149714 forward	87	34	PIPPSAPSERCN
SmelIDA1	Sme2.5_00993.1:18248..18481 forward	77	30	PIPPSAPSKRHN
SmelIDA2	Sme2.5_04429.1:34294..34539 forward	81	28	PIPPSAPSLRHN
SmelIDA3	Sme2.5_04724.1:40347..40592 forward	81	27	PIPASGPSRKHN
SmelIDA4	Sme2.5_06686.1:19811..20078 forward	85	25	PIPPSAPSDRCN
SmelIDA5	Sme2.5_08129.1:7336..7444 forward	102	34	PIPPSGPSKRHN
SmelIDA6	Sme2.5_09763.1:10983..11228 reverse	81	26	PVPPSAPSDRCN
CaIDA1	PepperUCD10Xch04:178438292..178438525 forward	77	27	PIPPSAPSKRHN
CaIDA2	PepperUCD10Xch06:176812434..176812673 reverse	79	29	PIPPSAPSKRHN
CaIDA3	PepperUCD10Xch11:6480406..6480714 forward	102	33	PIPPSGPSKRHN
CaIDA4	PepperUCD10Xch11:4624209..4624499 forward	96	35	PIPPSAPSKRHN
CaIDA5	PepperUCD10Xch11:6480457..6480714 forward	85	18	PIPPSGPSKRHN
CaIDA6	PepperUCD10Xch11:27920042..27920356 reverse	104	24	PIPPSEPSPRHN

*IDA*-like families of the *Nicotiana* species *N. sylvestris* and *N. tomentosiformis* consisted of 5 members, while in the allopolyploids *N. benthamiana* and *N. tabacum* these families are formed by 5 pairs of homeologs, with one exception corresponding to *NbenIDA4* whose homeolog pair was not found in the genomic screening. All *IDA*-like genes found in *Nicotiana* are new identifications, as the six members found in *S. melongena* and *C. annuum* and the seven members of the *S. tuberosum* family. In *S. lycopersicum*, five out of the eight *IDA*-like genes detected, members 1 to 5, were already described in [15] and named *SlIDA1–5*, while the other three peptides, *SlycIDA6–8*, are incorporated in the current work.

### Phylogenetic relationship among IDA-like prepropeptides in Solanaceae

**Fig. 1:**
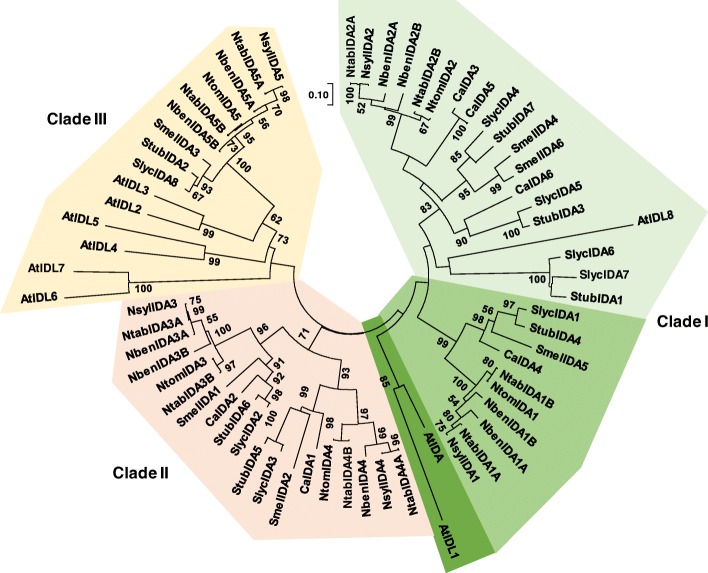
Unrooted circular phylogenetic tree of *IDA*-like prepropeptides of *Arabidopsis thaliana* and relevant species of the Solanaceae family such as *N. sylvestris*, *N. tomentosiformis*, *N. benthamiana*, *N. tabacum*, *S. lycopersicum*, *S. melongena*, *C. annuum* and *S. tuberosum*. Clade I shadowed in green color is divided in two subclades containing abscission-related genes (shadowed in lime green) and another one that grouped members of all eight Solanaceae species studied, as well as AtIDL8. The last subclade is subdivided in a group containing genes from *Nicotiana*, eggplant and pepper closed to SlIDA1, a tomato gene previously associated to abscission (shadowed in lime green) and other group (shadowed in light green) including from the *Nicotiana*, *Solanum* and *Capsicum* genera, with a small subdivision composed of AtIDL8 together with peptides from *S. lycopersicum* and *S. melongena*. Clade II (shadowed in light orange) includes exclusively Solanaceae genes. Clade III (shadowed in light gold) includes most of the Arabidopsis *IDA*-like family as well as *Nicotiana* and *Solanum IDA*-like genes. Bootstrap values are shown in each node

The phylogenetic relationships among the IDA-like members of the species of Solanaceae studied, in addition to those of Arabidopsis, are grouped in three major clades (Fig. 1). Clade I (shadowed in green colors) was divided in two subclades. The subclade shadowed in green contained the two Arabidopsis prepropeptides involved in floral organ abscission, AtIDA and AtIDL1 [1, 9]. The largest subclade grouped members of all eight Solanaceae species studied, as well as AtIDL8, the most divergent IDA-like peptide from Arabidopsis. In this subclade, Solanaceae members are further divided in two major groups. The group shadowed in lime green contained SlIDA1, the IDA-like member of tomato that has been associated with leaf abscission [15], other prepropeptides of potato (StubIDA4), eggplant (SmelIDA5) and pepper (CaIDA4) as well as the IDA1 members of the *Nicotiana* species under study. These were NsylIDA1 and NtomIDA1 of the diploid species *N. sylvestris* and *N. tomentosiformis*, respectively, and the two pairs of NtabIDA1 and NbenIDA1 homeolog prepropeptides corresponding to *N. tabacum* and *N. benthamiana*. The 5′-UTR regions and the predicted CDSs of all these *IDA1* genes from the genus *Nicotiana* showed high degree of conservation (see Additional files 1 and 3). The other group shadowed in light green included other prepropeptides from the *Nicotiana*, *Solanum* and *Capsicum* genera, with a small subdivision composed of AtIDL8 together with SlycIDA6, SlycIDA7 and StubIDA1 (Fig. 1). A second clade, clade II (shadowed in light orange), appeared to be limited to the Solanaceae family. This clade included prepropeptides from the *Nicotiana*, *Solanum* and *Capsicum* genera, but none from Arabidopsis, an observation suggesting that it might have diverged before the irruption of the Brassicaceae family 40 million years ago [33]. The third clade, clade III (shadowed in light gold), included AtIDL6 and AtIDL7, two IDA-like members of Arabidopsis that have been associated with processes different than cell separation, such as stress response [13]. The topology of the clade showed that there was a great diversification in Arabidopsis that generated at least six members, AtIDL2–7. It also included prepropeptides from the *Nicotiana* and *Solanum* genera, but none from *Capsicum*.

### *Cis*-acting regulatory elements in the promoter regions of the *N. benthamiana IDA*-like family

**Fig. 2:**
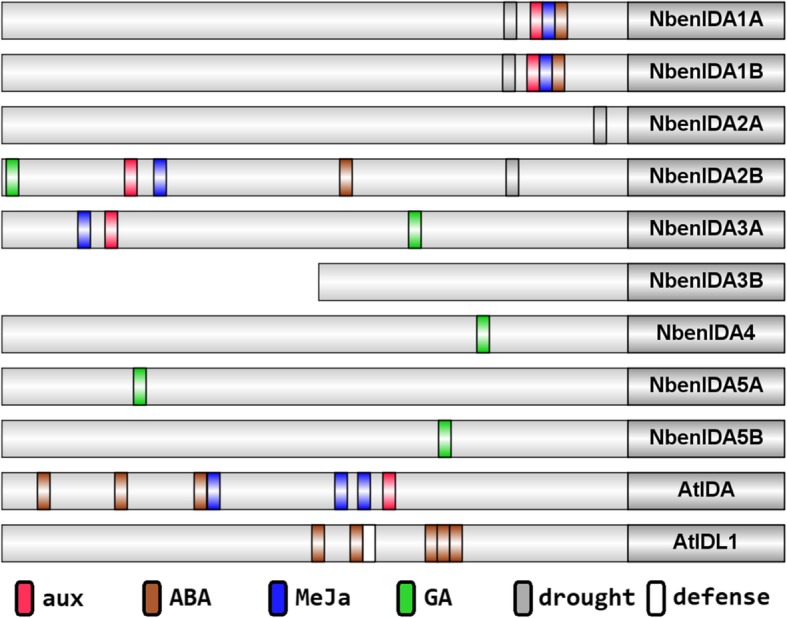
Schematic representation of *cis*-acting regulatory elements of the promoter regions of the *N. benthamiana IDA*-like gene family and Arabidopsis *AtIDA* and *AtIDL1*. Light grey boxes represent 1000 bp long promoter regions while dark grey boxes represent the 5′ part of the gene. In-silico searches of response elements to hormones related to abscission such as abscisic acid, methyl jasmonate, auxins or gibberellins, as well as response elements to biotic and abiotic stresses were performed. Response elements color scheme: red, auxins; brown, abscisic acid; blue, methyl jasmonate; green, gibberelins; grey, drought stress; white, defense response. The promoter region of *NbenIDA3B* is 493 bp long since the rest of the sequence is not available yet

Figure 2 shows a schematic representation of the *cis*-acting regulatory elements along 1000 bp of the 5′-UTR region of the *IDA*-like family members of *N. benthamiana* and *AtIDA* and *AtIDL1* of Arabidopsis. Searches for response elements to hormones related to ABA, methyl jasmonate (MeJa), AUXs or GAs, as well as response elements to biotic and abiotic stresses were performed. Interestingly, the pair of *NbenIDA1* homeologs contained similar promoter regions carrying response elements to ABA, MeJa and AUX as *AtIDA* in similar locations; these phytohormones have been involved in the abscission process in different ways [1, 34, 35]. The pairs of *NbenIDA1* and *NbenIDA2* homeologs also carry drought response elements in their promoter regions. On the other hand, *NbenIDA2B*, *NbenIDA3A*, *NbenIDA4*, and the pair of *NbenIDA5* homeologs are characterized by the occurrence of GA response elements (Fig. 2).

### Expression patterns of *IDA*-like and *HAE*-like genes in *Nicotiana benthamiana* during growth and abscission

**Fig. 3:**
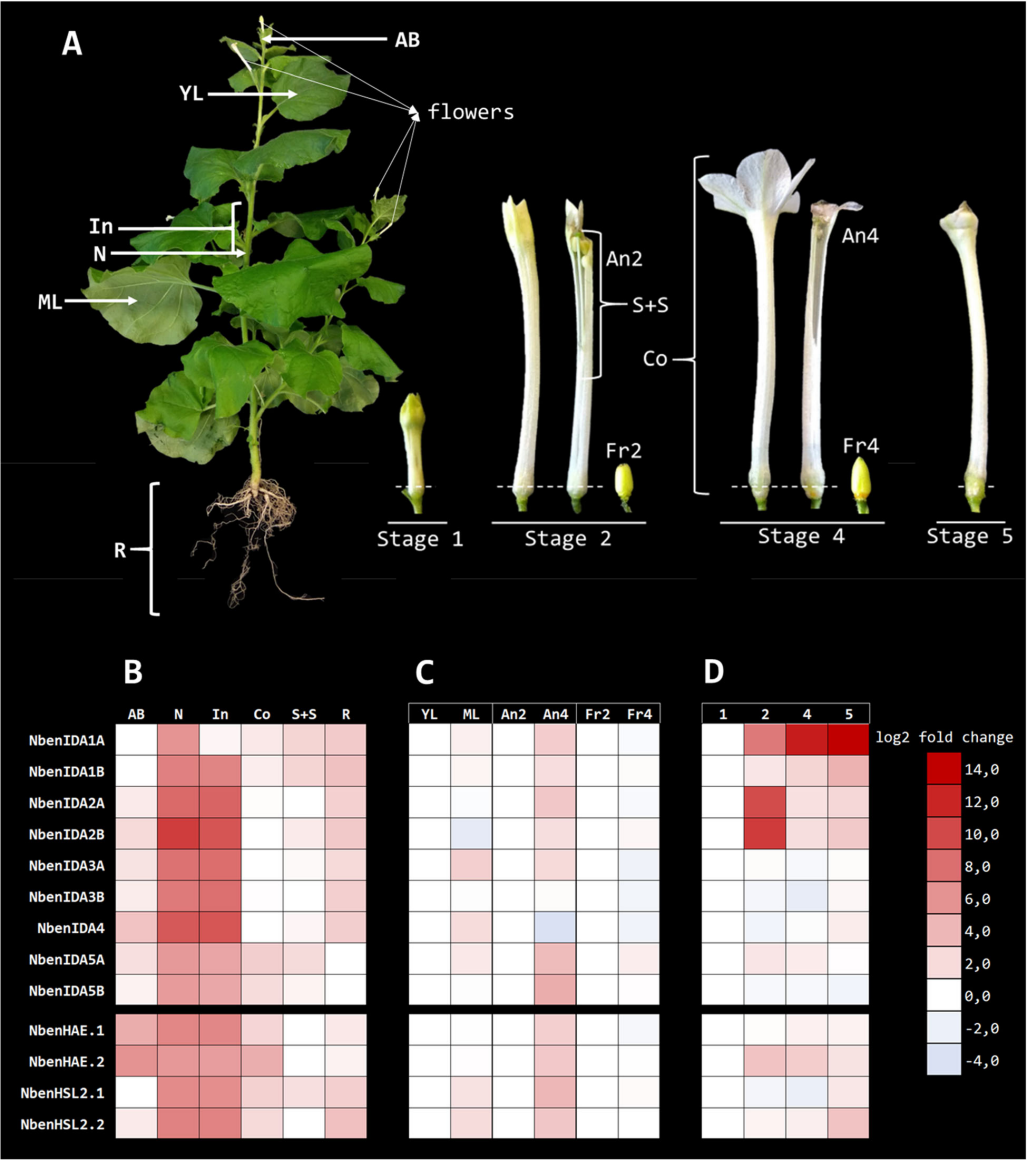
Expression patterns of *IDA*-like and *HAE*-like genes based on quantitative real-time PCR in several organs and tissues of *N. benthamiana* at different stages of corolla development. **a** Floral organs (sepals were removed), fruits and vegetative tissues utilized for gene expression analysis. The developmental stages of the corolla that were selected as a source of floral organs and fruits showed the corolla tube bulb above the calyx and the corolla limb closed (stage 1); fully elongated corolla tube and closed corolla limb and anthers (stage 2); open flower, corolla limb fully expanded and anthers dehiscent (stage 4); lobes of the corolla limb curl inwards, significant loss of turgor of the corolla tube which easily detached from the receptacle (stage 5). Dash lines mark the tissue collected from the base of the corolla. **b** Gene expression levels in apical buds (AB), nodes (N), internodes (In), whole corolla (Co), the ensemble formed by the style and the stigma (S + S) and roots (R) relative to the lowest expression level of each gene. **c** Gene expression patterns in leaves, anthers and fruits. Gene expression levels were relative to that at the earliest developmental stage in every organ (YL, young leaf; An2, anthers at stage 2; Fr2, fruit at stage 2, respectively). **d** Gene expression patterns in the corolla base during corolla developmental stages 1, 2, 4 and 5: Gene expression levels were relative to that at stage 1. Gene expression levels were normalized with respect to those of *NbenPP2A* gene, applying the 2^-ΔΔCt^ method. Relative gene expression levels (log2 fold change) are given next to the color scale column. Upregulation and downregulation of gene expression is shown by red and blue color, respectively. Gene expression raw and processed final data are shown in Additional File 5

Expression analysis of the family of *IDA*-like ligand peptides and their putative *HAE*-like receptors in different plant tissues of *N. benthamiana* are presented in Fig. 3. *HAE*-like receptors were identified through the analyses of the phylogenetic relationships between the *HAE*-like receptor-like kinases (RLKs) of Arabidopsis and *Nicotiana* (see Additional files 2 and 3). The plant material selected for gene expression analysis is shown in the panel A of Fig. 3. This plant material included different vegetative tissues of a plant in active growth (apical buds, young and mature leaves, nodes and internodes, and roots), as well as reproductive tissues (anthers, styles, stigmas, and fruits) at different developmental stages, including samples of the base of the flower corollas, a tissue that in tobacco (*N. tabacum*) has been shown to respond to the abscission process [25]. Panel B in Fig. 3 shows the expression of the *N*. *benthamiana IDA*-like and *HAE*-like homeologs in apical buds, nodes, internodes, the whole corolla, the ensemble formed by the stigma and the style and also in roots relative to the lowest expression level of each gene. Panel C in Fig. 3 shows the expression pattern of each *IDA*-like and *HAE*-like homeolog in leaves, anthers and fruits relative to that at the earliest developmental stage in every organ and panel D shows the expression patterns of each homeolog gene at the corolla base in developmental stages 2, 4 and 5 relative to the corolla developmental stage 1. Virtually all members of the *IDA*-like family of *N. benthamiana* were mainly expressed in nodes and internodes, although *NbenIDA1A* expression levels were not especially high in internodes (Fig. 3b). No changes in the expression patterns of *IDA*-like homeolog genes were observed in leaves and fruits (Fig. 3c) but the expression level for all of them except *NbenIDA3B* and *NbenIDA4* showed a tendency to increase between closed and dehiscent anthers (Fig. 3c). Interestingly, expression of both *NbenIDA1* homeologs at the base of the flower corolla increased with the stage of development of the tissue, in parallel to the progress of the abscission process (Fig. 3d). The expression pattern of *NbenIDA1B* was similar to that detected in *NbenIDA1A* although at a much more modest level. The expression levels of the *NbenIDA2* homeologs were transiently high in stage 2 when the corolla tube is fully elongated and the limb lobes are still closed to return later in stages 4 and 5 to almost the basal level of expression (Fig. 3d).

The highest expressions levels of the putative receptors of the IDA-like peptides, *NbenHAE.1*, *NbenHAE.2*, *NbenHSL2.1* and *NbenHSL2.2*, were also registered in nodes and internodes (Fig. 3b). Additionally, their expression levels also showed a tendency to increase between closed and dehiscent anthers (Fig. 3c) and a slight increase was observed at the corolla base associated with corolla development (Fig. 3d).

### Expression patterns of *IDA*-like genes in *Nicotiana benthamiana* during water stress

**Fig. 4:**
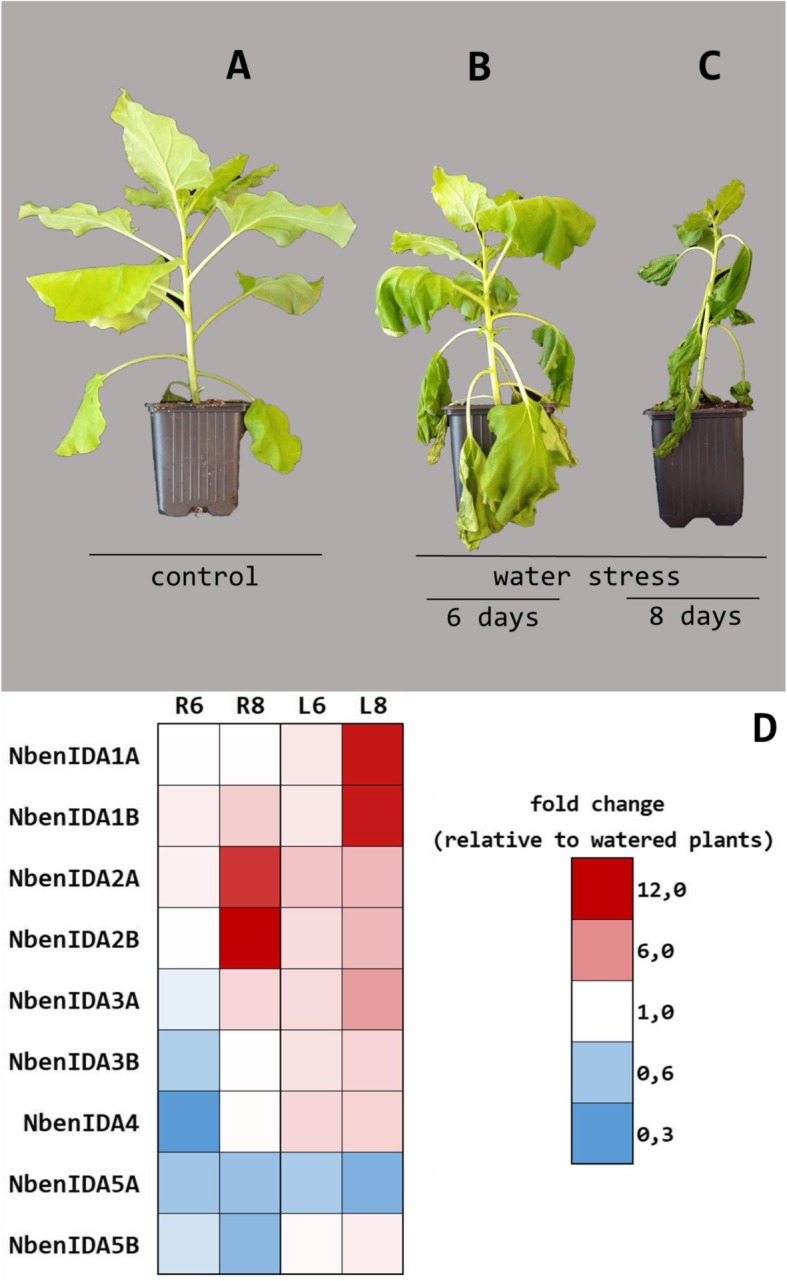
Expression patterns of *IDA*-like genes based on quantitative real-time PCR in control and water stressed plants of *N. benthamiana*. General appearance of a well-watered plant (**a**) and plants subjected to water stress during 6 days (**b**) and during 8 days (**c**). Expression patterns in roots (R) and mature leaves (L) of water stressed plants during 6 or 8 days (**d**). Expression levels were calculated through the 2^-ΔΔCt^ method, normalized to that of *NbenPP2A* gene and relative to gene expression in control (watered) plants. For each stressed organ the appropriate reference was used (well-watered leaf or root). Expression levels relative to watered plants (fold change) are given next to the color scale. Red, white and blue colors indicate, respectively, gene induction (values over 1), unchanged (values close to 1) and repression (values under 1) all of them regarding to that of *NbenPP2A* gene in the relevant watered (control) organ. Gene expression raw and processed final data are shown in Additional File 5

The presence of drought response elements in the promoter regions of some particular *IDA*-like members, e.g. *NbenIDA1A*, *NbenIDA1B*, *NbenIDA2A* and *NbenIDA2B* (Fig. 2), suggested that their expression might be regulated by the water status of the plant. Therefore, we exposed actively growing plants of *N. benthamiana* to 6 (mild stress) and 8 (severe stress) days of water stress and the expression levels of all members of the *IDA*-like family in axillary buds, roots and leaves were determined (Fig. 4). While no differences in gene expression were found in axillary buds, those of the pair of *NbenIDA1* homeologs dramatically increased in leaf blades of plants subjected to severe water stress. In contrast, this condition resulted in higher increases in transcripts belonging of both *NbenIDA2* homeologs in roots, indicating differential roles of this gene family in response to water stress. Changes in the expression of the rest of genes were of minor relevance although it is worth to mention that these members tended to repress their expression levels in roots of plants subjected to water stress, although *NbenIDA5A* expression was also reduced in stressed leaves.

## Discussion

*IDA*-like genes were searched in relevant genera of the Solanaceae family including several species of *Nicotiana* (*N. sylvestris*, *N. tomentosiformis*, *N. tabacum* and *N. benthamiana*), and other crops of agronomic interest such as tomato (*Solanum lycopersicum*), potato (*S. tuberosum*), eggplant (*S. melongena*) and pepper (*Capsicum annuum*) (Table 1). This gene family was identified in a large number of Angiosperms [17] and their members contained a signal peptide targeting the protein to the apoplast through the secretory pathway and a conserved C-terminal part, the PIP motif (Additional file 4). The presence of a signal peptide in the sequence of all identified genes suggests a mechanism of posttranslational maturation in the apoplast similar to that described in Arabidopsis, where the prepropeptide is proteolytically processed by subtilisin-like serine proteinases to yield a bioactive peptide [36].

The reduced size of the mature IDA-like peptides (an alignment of the complete coding sequences of these genes can be seen in Additional file 4), precluded the study of the phylogenetic relationships based on these premises and, therefore, a circular phylogenetic tree was generated using complete sequences encoding prepropeptides including a signal peptide and a variable region (Fig. 1). This tree shows that the Arabidopsis *IDA*-like gene family exhibits a higher degree of diversification than the Solanaceae genera studied, with the exception of *Nicotiana*. The two Arabidopsis prepropeptides related to abscission, AtIDA and AtIDL1 [1, 34], nested in a small clade grouped with a larger clade including many *IDA*-like members from the different species of Solanaceae. This large clade was divided in two subgroups in one of which was nested the tomato *SlycIDA1*, associated with abscission [15], and its homolog genes in *S. melongena*, *C. annuum*, *S. tuberosum* and in the four *Nicotiana* species studied. The genus *Nicotiana* originates in South America and its members have spread over four continents. This genus consists of diploid species and several allopolyploid species of different ages and paternity. Thus, cultivated tobacco (*N. tabacum*) is an allotetraploid (2n = 4x = 48) representing a hybridization event involving the diploid species *N. sylvestris* (2n = 2x = 24) and *N. tomentosiformis* (2n = 2x = 24) as their female and male parentals, respectively. This hybridization event is believed to have occurred recently, less than 200,000 years ago [37, 38]. The fact that all homeologs of the *IDA*-like family of *N. tabacum* have corresponding counterparts in *N. sylvestris* and *N. tomentosiformis* (Table 1 and Fig. 1) is in line with this observation (Fig. 1).

In contrast to tobacco, *N. benthamiana* has been described as an ancient allotetraploid whose polyploidy level (2n = 4x = 38) likely evolved through genome re-arrangements and fractionation giving rise to a remarkable descending dysploidy [38]. Parentals of *N. benthamiana* are unknown, although it is believed that it comes from a hybridization event that occurred > 10 Myr ago between species belonging to the *Sylvestres* and *Noctiflorae* sections of *Nicotiana* [38]. Two homeologs were identified for most of the analysed genes in our work, except for *NbenIDA4*. Genomic responses to polyploidy are complex in *Nicotiana* species, ranging from small to large genome re-sizing depending on the polyploid age and the similarity of parental genome donors [38]. Reduction in the number of chromosomes in *N. benthamiana* strongly suggests a considerable genome downsizing, probably as a consequence of being an older polyploid. The size reduction involves 1 Gb in length relative to tobacco (4.5 Gb genome size of *N. tabacum*, 3.5 Gb *N. benthamiana*). Therefore, we suggest that gene loss might explain the absence of a second copy of *NbenIDA4* in our data, rather than a misrepresentation in the draft assembly of the genome used for the analysis. Interestingly, several genetic studies estimated that the genome of *N. tabacum* had lost DNA from its progenitors since polyploidization and that this genomic loss was greater and biased towards the genome of the male parental *N. tomentosiformis* [38–41]. Therefore, we also think that similar biased gene loss may have happened in *N. benthamiana* involving the copy of *NbenIDA4* belonging to the parent of the *Noctiflorae* section of *Nicotiana*.

The analyses of the *cis*-acting regulatory elements in the 5′-UTR regions of the *N. benthamiana IDA*-like family and Arabidopsis *AtIDA* and *AtIDL1* (Fig. 3) failed to identify ethylene response elements, in agreement with the idea that *IDA*-like genes regulating the abscission process are not directly dependent upon ethylene [42, 43]. In contrast, the presence of response elements to AUXs, ABA, MeJa and GAs in the promoter regions of the *IDA*-like family members of *N. benthamiana* and *AtIDA* and *AtIDL1* suggests that these phytohormones might play a role in regulating the expression of these genes. The occurrence of functional indole-3-acetic acid (IAA) signaling in the abscission zone during organ separation, for instance, has been demonstrated by Basu and co-workers [44]. It has also been determined that ABA and MeJa have abscission-promoting effects, while the role of GAs is not entirely clear [6, 45]. However, it has been shown in citrus that flower pollination increased bioactive gibberellin A1 (GA_1_) levels and reduced ovary abscission and that the treatment of unpollinated ovaries with GA_3_ also suppressed ovary abscission [46, 47].

Our bioinformatic analyses also indicated that the coding and promoter sequences of the pair of *NbenIDA1* homeologs are highly similar and that promoters share the same hormonal response elements in similar positions, in addition to the same drought response element (Fig. 2). High conservation of the coding and promoter sequences of *IDA1* duplicated genes in *N. sylvestris, N. tomentosiformis* and *N. tabacum* (see Additional file 3) suggests that they may be very important in the regulation of cell separation processes and response to stressful conditions. Furthermore, the pair of *NbenIDA2* homeologs also contains drought stress response elements in their promoter regions (Fig. 2). Likewise, the coding and promoter sequences of the *IDA1* genes in *N. attenuata*, *N. sylvestris, N. tomentosiformis, N. benthamiana* and *N. tabacum* are very similar and have the same response elements in the same positions in their promoters, except *N. attenuata* (Additional file 1).

The results described above (Fig. 1) suggested that in *N. benthamiana,* NbenIDA1A and NbenIDA1B peptides may be involved in the abscission process. This suggestion is also supported by the gene expression patterns found at the corolla base of the flowers during the process of natural abscission (Fig. 3d). Similarly, there seems to be a correlation between the expression of the *IDA*-like genes and that of their putative receptors of the *HAE*-like family, *NbenHAE.1*, *NbenHAE.2* and *NbenHSL.2.2* (Fig. 3d), that also increased during the last phases of the corolla abscission.

As described for *IDA*-like families in other species [12], the different members of the *N*. *benthamiana* family are also expressed in multiple plant tissues (Fig. 3). This is not a surprise since the IDA-like signaling peptides, as cell-to-cell communication elements, function in several cell separation events, including lateral root emergence and root cap sloughing [10, 11]. Interestingly, in plants of *N. benthamiana* actively growing, the highest expression level of most members of the *IDA*-like family was found in nodes and internodes. It is worth mentioning that the promoter regions of *NbenIDA2B*, *NbenIDA3A*, *NbenIDA4*, *NbenIDA5A* and *NbenIDA5B* genes contain GAs response elements, and that these hormones are pivotal regulators of stem growth [48]. Moreover, all *HAE*-like genes analyzed also show higher expression levels in nodes and internodes, in parallel with the pattern observed for the *IDA*-like genes. These expression patterns might be linked to the formation of vascular bundles and to the cell elongation and division associated with the process of stem elongation implying cell wall remodeling.

The occurrence of cis-acting elements related to the drought response in the two pairs of *NbenIDA1* and *NbenIDA2* homeologs (Fig. 2) also suggested to test the response of the *IDA*-like genes to water stress conditions. In the experiment reported in Fig. 4 it is clearly observed that the first pair of homeologs was highly expressed in leaves from *N. benthamiana* plants severely stressed while in roots, the genes that responded to water deficit were the members of the second pair. Furthermore, these two pairs of homeologs are phylogenetically close to *AtIDA* and *AtIDL1*, two Arabidopsis genes that are induced under abiotic stress conditions [12].

Our gene expression data showed that while most pairs of homeologs showed similar expression patterns, some of them exhibited divergence in certain organs and tissues studied (see Fig. 3). This might well be linked to the frequent observation that some duplicated genes, after a whole genome duplication event, evolve to undertake different functions or partition the function of the ancestral gene in a process of subfunctionalization. This process can include epigenetic, coding sequence or promoter modifications that alters regulatory mechanisms (e.g. silencing) and give rise for example to differential level of expression or tissue specificity. Subfunctionalization becomes more relevant when gene dosage is not an adaptive advantage for the polyploid [49]. Therefore, our gene expression data might be revealing a putative subfunctionalization for the homeolog pairs *NbenIDA1* at the corolla base and *NbenIDA3* in leaves and anthers. We took special care in primer specificity during qPCR assays in order to distinguish between both homeologs, since gene expression artifacts may be recurrent among genes derived from genome duplicated areas due to high sequence similarity.

It has been recently observed that IDA signaling peptides can certainly regulate important developmental processes as well as fundamental plant responses to environmental conditions [13]. Our data indicate that in the allopolyploid *N. benthamiana*, the two pairs of *NbenIDA1* and *NbenIDA2* homeologs are differentially involved in the responses to drought stress while only *NbenIDA1* homeologs are apparently implicated in the natural process of corolla abscission. These data suggest that IDA-like signaling peptides can play different biological roles in various tissues and under distinct abiotic conditions.

## Conclusions

We have investigated the *IDA*-like and *HAE*-like gene families of different Solanaceae species, *S. lycopersicum, S. melongena, C. annuum, S. tuberosum,* and four species of the genus *Nicotiana*, *N. sylvestris, N. tomentosiformis, N. benthamiana,* and *N. tabacum* and determined their phylogenetic relationships. In the allopolyploid *N. benthamiana,* specific analyses of the the *cis*-acting regulatory sequences and the examinations of the gene expression patterns of the *IDA*-like family have identified putative candidate *IDA*-like genes implicated in corolla abscission and in the response to water stress. The results suggest that the pair of *NbenIDA1* homeologs are both involved in the natural process of corolla abscission. Interestingly, they also show specific differential expression under water stress conditions. *NbenIDA1* homeologs are highly expressed in stressed leaves while *NbenIDA2* homeologs, especially *NbenIDA2B*, are highly expressed in stressed roots. In addition, nodes and internodes are the tissues with the highest expression of the *IDA*-like and *HAE*-like genes in normal active growing plants, suggesting that these peptides are also essential during stem growth and development. These results add new evidence that the functional module formed by IDA-like peptides and its receptor kinases as defined in Arabidopsis, may be conserved in Solanaceae.

## Methods

### Retrieval and sequence analysis

The EPIP motif of AtIDA (FGYLPKGVPIPPSAPSKRHNSFVNSLPH) was used to identify the *IDA*-like members of the selected Solanaceae species (*N. sylvestris, N. tomentosiformis, N. tabacum, N. benthamiana, Solanum lycopersicum, S. tuberosum, S. melongena and Capsicum annuum*) by tBLASTn and BLASTp inquiries in the Sol Genomics [50] web platform (https://solgenomics.net/tools/blast/), depending on the databases status. “N.sylvestris Genome”, “N.tomentosiformis Genome”, “N.tabacum BX Genome”, “N.benthamiana v1.0.1”, “Tomato ITAG release 3.20”, “Potato PGSC DM v3 scaffolds”, “Eggplant draft genome (release 2.5.1)” and “*Capsicum annuum* UCD10X genome chromosomes (v1.0)” databases [30, 51–55] were used, respectively. Arabidopsis AtHAE, AtHSL1 and AtHSL2 protein sequences were retrieved from Phytozome v12.1, TAIR10 database and were used to identify the *HAE*-like members of the selected Solanaceae species in the same way as described above. Newly identified genes were named numerically, adding an “A”, “B”, “.1” or “.2” termination to the *IDA*-like or *HAE*-like gene pairs for the allopolyploids *N. tabacum* and *N. benthamiana*.

Sequence alignments were performed through MEGA7 software [56] using the ClustalW algorithm with default parameters (DNA Data Bank of Japan, DDBJ; http://clustalw.ddbj.nig.ac.jp/). Phylogenetic trees were created using the Neighbor-Joining method [57] using 1000 bootstrap replicates. The trees are drawn to scale, with branch lengths in the same units as those of the evolutionary distances used to infer the phylogenetic trees. The evolutionary distances were computed using the Poisson correction method [58] and are in the units of the number of amino acid substitutions per site. All ambiguous positions were removed for each sequence pair (pairwise deletion option).

Peptide localization prediction and presence of signal peptides in the IDL amino acid sequences were analyzed using the TargetP [31] and SignalP-5.0 [32] services. Up to 1000 base pairs of promoter regions upstream of the start codon of the IDL genes of the *Nicotiana* species available in Sol Genomics databases were retrieved and submitted for cis-acting regulatory element analysis in PlantCARE [59]. Schematic representations of regulatory elements of the promoter sequences where created using IBS1.0.3 software [60]. All DNA and protein sequences, as well as the localization of the sequences in the genomes in SolGenomics and the sequence IDs are contained in Additional file 7.

### Plant materials and growth conditions

*N. benthamiana* seeds were obtained from Dr. José Guerri and Dr. Karelia Velázquez of the Centro de Protección Vegetal y Biotecnología (IVIA, Moncada, Spain). The seeds were germinated on nutrient soil and transplanted individually in small pots with an artificial potting mix (50% vermiculite and 50% peat moss) in a plant growth chamber at 20/24 °C (night/day), 60% relative humidity and a 16/8-h light/dark regime. Water stress was induced by not watering the plants for 6 and 8 days for mild and severe stress conditions respectively.

### RNA extraction

Basal portion of the corollas at different flower development stages as well as the rest of tissues used in gene expression analysis were manually collected from the plants and frozen with liquid nitrogen. Tissues were grinded using Thomas Scientific’s Liquid Nitrogen Cooled Mortar. Total RNA was extracted using Macherey-Nagel’s NucleoSpin® RNA Plant, following the manufacturer’s instructions. cDNA was synthesized from the RNA extraction using Thermo Fisher Scientific’s SuperScript™ II Reverse Transcriptase, following the manufacturer’s instructions.

### qPCR analysis

Quantitative PCR analysis were performed using LightCycler® FastStart DNA MasterPLUS SYBR Green I reaction mix and a LightCycler 2.0 instrument (Roche, Basel, Switzerland) using gene-specific primers designed based on transcriptome sequences using the Primer3Plus software [61]. Primer pairs are listed in Additional file 6. Specificity of all the primer pairs used in this work was assessed by primer BLAST and melting curve analysis [62, 63]. The fluorescence intensity data was obtained through LightCycler Software version 4.1. The *N. benthamiana* housekeeping gene *NbenPP2A* [64] was used for normalization in all qPCR reactions carried out in this work. Three biological replicates were run for assessing the expression values of each gene. The averaged expression values were obtained in the form of Ct (cycle threshold) and all the analyzes were performed through 2^-ΔΔCT^ method.

For gene expression relative quantification of *IDA*-like and *HAE*-like genes in different plant tissues and organs in wild type plants, every gene expression was normalized respect to *NbenPP2A* expression. In apical buds, nodes, internodes, corolla, style and stigma, and root tissues, gene expression values were relative to the lowest expression value of each gene in the relevant tissue or organ, within primer sets. In leaf, anthers, fruits and corolla base tissues, gene expression values were relative to the expression value of the earliest developmental stage relevant for that tissue (young leaf, anthers and fruits in stage 2, and corolla base of a stage 1 flower, respectively) within primer sets. Units were represented as the log2 of the fold change. In the case of leaf, anthers, fruits and base of the corolla tissues, red color indicates that the gene is upregulated (values over 0); white, that remain unchanges (values close to 0); and blue, that the gene is downregulated (values under 0); all respect to *NbenPP2A* expression in the corresponding tissue in its earliest developmental stage.

In the water stress experiment, *NbenPP2A* was also used as a housekeeping gene for normalization, and watered tissue expression values as a relative reference, thus constituting our control conditions. Conditions were appropriate for each measurement, using the corresponding control tissue (leaf or root of watered plants) as a relative reference. Units were represented as fold change. Red color indicates that the gene is upregulated (values over 1); white, that remain unchanged (values close to 1); and blue, that the gene is downregulated (values under 1); all respect to *NbenPP2A* expression in the corresponding watered (control) tissue.

## Supplementary information

**Additional file 1: **Nicotiana *IDA1* promoters and coding sequences alignment.pdf. Alignment of the 5′-UTR sequences (500 bp) and the CDS of *NbenIDA1A*, *NbenIDA1B*, *NtabIDA1A*, *NtabIDA1B*, *NsylIDA1* and *NtomIDA1* genes. Start codon is highlighted in green, and cis-acting regulatory elements are highlighted as follows: brown line, abscisic acid; blue line, methyl jasmonate; red line, auxins; grey line, drought.

**Additional file 2: **Solanaceae HSL family.pdf. *HAE*-like gene families in species of the Solanaceae family, (genome localization from different Sol Genomics Network databases [50]).

**Additional file 3: **HAE-like peptides phylogenetic tree.pdf. Circular phylogenetic tree of HAE-like peptides of *Arabidopsis thaliana* and several species of the Solanaceae family (*N. benthamiana, N. tabacum, N. sylvestris, N. tomentosiformis, S. lycopersicum, S. tuberosum, S. melongena and C. annuum)*.

**Additional file 4: **Alignment IDA-like prepropeptides.pdf. Sequence alignment of IDA-like peptides from several species of the Solanaceae family (*N. benthamiana, N. tabacum, N. sylvestris, N. tomentosiformis, S. lycopersicum, S. tuberosum, S. melongena and C. annuum*) and from Arabidopsis. The CINEMA color scheme is used to inform about the chemical nature of the amino acid residues in the EPIP domain (blue, polar positive; red, negative; green, neutral; white, non-polar aliphatic; purple, ocher and yellow, aromatic residues).

**Additional file 5:** Gene expression raw and final processed data.xlsx.

**Additional file 6:** qPCR primers.pdf. Primers used for quantitative PCR analysis.

**Additional file 7:** Solanaceae IDA-like and HAE-like gene families.xlsx. The DNA and protein sequences, as well as the localization of the sequences in the genomes in SolGenomics and the sequence IDs are contained in this file.

## Data Availability

All data generated or analysed during this study are included in this published article and its supplementary information files.

## Abbreviations

ABA: Abscisic acid
AUX: Auxins
GA: Gibberellin
HAE: HAESA
IDA: INFLORESCENCE DEFICIENT IN ABSCISSION
