# Supplementary material for: Differential expression of IDA (INFLORESCENCE DEFICIENT IN ABSCISSION)-like genes in Nicotiana benthamiana during corolla abscission, stem growth and water stress
Source: BMC Plant Biol. 2020 Jan 20;20:34. doi: 10.1186/s12870-020-2250-8 (PMC6971993; doi:10.1186/s12870-020-2250-8)
Supplement: Supplementary file 1 — Additional file 1: Nicotiana IDA1 promoters and coding sequences alignment.pdf. Alignment of the 5′-UTR sequences (500 bp) and the CDS of NbenIDA1A, NbenIDA1B, NtabIDA1A, NtabIDA1B, NsylIDA1 and NtomIDA1 genes. Start codon is highlighted in green, and cis-acting regulatory elements are highlighted as follows: brown line, abscisic acid; blue line, methyl jasmonate; red line, auxins; grey line, drought. [file 12870_2020_2250_MOESM1_ESM.pdf]

```
NbenIDA1A      ----------CCACAAGCTTCTGTCAATTTCTGAC------GTGGAAGAGTTTACTTTTT 44
NtabIDA1B      ------------------TCAGTTGACTTGCAAA---AGAAAAGAAAAAAAATACTATTT 39
NtomIDA1       ------------------TCAGTTGACTTGCAAA---AGAAAAGAAAAAAAATACTATTT 39
NbenIDA1B      GGGAAACTCATGAGAAAATCCCTTAAGTTG-----------ACTTAAAAAAAACATTATT 49
NtabIDA1A      ------CTCATAAGAAAATCCCTTCAGTTGACTTGAAAAAGAAAAAGAAAAACATTATTT 54
NsylIDA1       -------TCATAAGAAAATCCCTTCAGTTGACTTGAAAAAGAAAAAGAAAAACATTATTT 53
                                 *    * * **                *  *      *  **

NbenIDA1A      -TCC-------AAATTTTAGCTGTTCTGGTCAAAGGGTTCTATACAGTATAATACCTAAA 96
NtabIDA1B      AAACAGGAAATAACTTTAGTCACCTCCGATCCGAT----CTTTACACCCAACTACATTAA 95
NtomIDA1       AAACAGGAAATAACTTTAGTCACCTCCGATCCGAT----CTTTACACCCAACTACATTAA 95
NbenIDA1B      CAACAGGAAATAACCTTAATCACTT-----CCGAT----ATTTATATCAAATTATATTAA 100
NtabIDA1A      CAACAGGAAATAACCTTAGCCACCT-----CAGAT----ATTTACACCAGATTATATTAA 105
NsylIDA1       CAACAGGAAATAACCTTAGCCACCT-----CAGAT----ATTTACACCAGATTATATTAA 104
                  *       **  **   *   *     *  *      * ** *    * **  * **

NbenIDA1A      CTTAAAGTAAGATTATTATCTAATTTTAGTAAAACTGGTTTGATTAAAATATTATTATGA 156
NtabIDA1B      TTTATCATAA-TTAATATTA-------TAAAAATATTGACAAATGTACTAACTAGCTATA 147
NtomIDA1       TTTATCATAA-TTAATATTA-------TAAAAATATTGACAAATGTACTAACTAGCTATA 147
NbenIDA1B      TTTATATTAG-TTA-ATATT-------AAAAAATATTGATAGATGTACGGACTAGCTATA 151
NtabIDA1A      TTTATATTAA-TTA-ATATT-------AAACAATATTGACAGATGTACTGACTAGCTATA 156
NsylIDA1       TTAATATTAA-TTA-ATATT-------AAAAAATATTGACAGATGTACTGACTAGCTATA 155
                * *   **   *     *            **  * *    **  *   * **     *

NbenIDA1A      ACGGGTAAACCTAAATTTATGCATAAAGATAA--------CTAAATAGTGTTCATTGGTT 208
NtabIDA1B      ATTCAGTGACAAAAGTTTATGCATAAAAATATTAATACATATATTAAGTATTCAAT-GGT 206
NtomIDA1       ATTCAGTGACAAAAGTTTATGCATAAAAATATTAATACATATATTAAGTATTCAAT-GGT 206
NbenIDA1B      ATTCAGTGACAAAAGTTTATGCATAAAAATAC------ATAAAGCAAATATTTATT-GGT 204
NtabIDA1A      ATTCAGTGACAAAAGTTTATGCATAAAAATAC------ATAAAGCAAGTATTTATT-GGT 209
NsylIDA1       ATTCAGTGACAAAAGTTTATGCATAAAAATAC------ATAAAGCAAGTATTTATT-GGT 208
               *       **  ** ************ ***           *   * * ** * * * *

NbenIDA1A      CGATGTAAATATCGTGTCAAATT-TTTTTCCTTAAAACATATACTCCATAAGAAGAAAAC 267
NtabIDA1B      TGATGTAAACATCGTGTATGTGTGTAAAATTTTTCCCCTTAAAACAGATAAGAAGAAAAC 266
NtomIDA1       TGATGTAAACATCGTGTATGTGTGTAAAATTTTTCCCCTTAAAACAGATAAGAAGAAAAC 266
NbenIDA1B      TGTTATAAATATCGTGTATGTGTGTAAAATTTTTCTCTT-AAAACATATAACAAGAAAAC 263
NtabIDA1A      TGGTACAAGCATCGTGTATGTGTGTAAATTTTTTCTCTT-AAAACATATAAGAAGAAAAC 268
NsylIDA1       TGGTACAAGCATCGTGTATGTGTGTAAATTTTTTCTCTT-AAAACATATAAGAAGAAAAC 267
                * *  **  *******     * *      **       * *    **** ********

NbenIDA1A      GCCGGCGTTTATTCCAAATCATTTAATATATATTTTTTCTTATCAGTTGACATTTATCAA 327
NtabIDA1B      GCCGGCATTTATTCCAAATCATT-TAATATAGTTTTTTCTTATCAGTTGACATTTATTAA 325
NtomIDA1       GCCGGCATTTATTCCAAATCATT-TAATATAGTTTTTTCTTATCAGTTGACATTTATTAA 325
NbenIDA1B      GCCGGCGTTTATTCCAAATCATTTA-ATAATTTTTTTTCTTATCAGTTGACATTTATTAA 322
NtabIDA1A      GCCGGCGTTTATTCCAAATCA-TTTAATATATTTTTTTCTTATCAGTTGACATTTATTAA 327
NsylIDA1       GCCGGCGTTTATTCCAAATCAATTTAATATATTTTTTTCTTATCAGTTGACATTTATTAA 327
               ****** ************** *         ************************* **
```

drought

```
NbenIDA1A      AGAATTGTTTAATTTAGTCGTTTGACGTGTGAATCACTTAACCTTTATTGCCGAACTCTT 387
NtabIDA1B      AGAATTGTTTAATACAGTCGTTTGACGTGTGAATCACTTAACCTTTTTTGCCGAACTCTT 385
NtomIDA1       AGAATTGTTTAATATAGTCGTTTGACGTGTGAATCACTTAACCTTTTTTGCCGAACTCTT 385
NbenIDA1B      AGAATTGTTTAATTTAGTCGTTTGACGTGTGAATCACTTAACCTTTTTCGCCGAACTCTT 382
NtabIDA1A      AGAATTGTTTAATTTAGTCGTTTGACGTGTGAATCACTTAACCTTTTTTGCCGAACTCTT 387
NsylIDA1       AGAATTGTTTAATTTAGTCGTTTGACGTGTGAATCACTTAACCTTTTTTGCCGAACTCTT 387
               *************  ******************************* * ***********
```

auxins

methyl jasmonate

abscisic acid

```
NbenIDA1A      TGCTTCCCCATGCACATACATTTGCACATATATATAAC-----CCTACTTCTTTTGCCTA 442
NtabIDA1B      TACTTCCCCATGCACATACATTTCCTCATATATATAT---AACCCTACTTCATTTACTTA 442
NtomIDA1       TACTTCCCCATGCACATACATTTCCTCATATATATAT---AACCCTACTTCATTTACTTA 442
NbenIDA1B      TACTTCCCCATGCACATATATTTGCTCATATATATAACCCAACCCTACTTCATTTACTTA 442
NtabIDA1A      TGCTTCCCCATGCACATACATTTGCACATATATATAACCC-----TACTTCATTTACTTA 442
NsylIDA1       TGCTTCCCCATGCACATACATTTGCACATATATATAACCC-----TACTTCATTTACTTA 442
               * **************** **** * **********         ****** *** * **
```

```
NbenIDA1A      AAAATTAAACCCAAGTTCAAAAAACCCTATTAGAAATTCAAGAAAATCCTCTCAGTTAAT 502
NtabIDA1B      AAAATTAAACCCAAGTTCAAAAAACCCTATTAGAAATTCAAGAAAACCCTCTCAATTAAT 502
NtomIDA1       AAAATTAAACCCAAGTTCAAAAAAGCCTATTAGAAATTCAAGAAAACCCTCTCAATTAAT 502
NbenIDA1B      AAATATAAACCCAAGTTCAAAAATCCCTATTAGAAATTCAAGAAAATCCTCTCAATTAAT 502
NtabIDA1A      AAAATTAAACCCAAGTTCAAAAAACCCTATTAGAAATTCAAGAAAATCCTCTCAATTAAT 502
NsylIDA1       AAAATTAAACCCAAGTTCAAAAAACCCTATTAGAAATTCAAGAAAATCCTCTCAATTAAT 502
               ***  ******************  ********************* ******* *****

NbenIDA1A      GGCTTCCTCCTCCTC---------------TTCCTCTTCTTCTTCTAAAAATAAAACCCC 547
NtabIDA1B      GGCCTCCTCCTCCTCCTC------------TTCCTCTTCTTCTTCTAAAAATAAAACTCT 550
NtomIDA1       GGCCTCCTCCTCCTCCTC------------TTCCTCTTCTTCTTCTAAAAATAAAACTCT 550
NbenIDA1B      GGCCTCCTCCTCTT------------------CCTCTTCTTTTTCTAAAAACAAAACCAT 544
NtabIDA1A      GGCCTCCTCCTCCTCCTCTTCCTCTTCCTCTTCCTCTTCTTCTTCTAAAAATAAAACCCT 562
NsylIDA1       GGCCTCCTCCTCCTCCTCTTCCT------CTTCCTCTTCTTCTTCTAAAAATAAAACCCT 556
               *** ******** *                  ********* ********* *****

NbenIDA1A      TTTTTACTTAATTTGTTTGATTCTTGCCATTTCTTTTCTTGTTGGTTATGGAGTTGAAGC 607
NtabIDA1B      TTATTACTTAATTTGTTTGATTCTTGCCATTTCTTTTCTTCTTGGTTATGGAGTCGAAGC 610
NtomIDA1       TTATTACTTAATTTGTTTGATTCTTGCCATTTCTTTTCTTCTTGGTTATGGAGTCGAAGC 610
NbenIDA1B      TTATTATTTAATTTGCTTGATTCTTGCCATTTCTTTTCTTCTTGATTATGGAGTTGAAGC 604
NtabIDA1A      TTATTACTTAATTTGTTTGATTCTTGCCATTTCTTTTCTTGTTGGTTATGGAGTTGAAGC 622
NsylIDA1       TTATTACTTAATTTGTTTGATTCTTGCCATTTCTTTTCTTGTTGGTTATGGAGTTGAAGC 616
               ** *** ******** ************************ *** ********* *****

NbenIDA1A      AAGACCAGGAAGAATGATAAAGGAGGAAGAAGAAGCCAATTCAAGAATATTTTCAACACA 667
NtabIDA1B      AAGACCAATC-------------------GAAGAAGCTAATTCAAGAATATTTTCATCACA 652
NtomIDA1       AAGACCAATC-------------------GAAGAAGCTAATTCAAGAATATTTTCATCACA 652
NbenIDA1B      AAGACCAGGGAGAATGATAATGGAGGGAAAAAAAGCAAATTCAAGAATATTTTCAACACA 664
NtabIDA1A      AAGACCAGGGAGAATGATAATGGAGGAAGAAGAAGCAAATTCAAGAATATTTTCAACACA 682
NsylIDA1       AAGACCAGGGAGAATGATAATGGAGGAAGAAGAAGCAAATTCAAGAATATTTTCAACACA 676
               *******                      ** ****  ****************** ****

NbenIDA1A      ACATTTGAAGGCATACAGAAAAGAAAATGCATACAAAACAGAAAATTTGGTATTTACTAT 727
NtabIDA1B      ACATTTGAAGGTATACAGAAAAGAGAATGCATACAAAACAGAAAATTTGCTATTTACTAT 712
NtomIDA1       ACATTTGAAGGTATACAGAAAAGAGAATGCATACAAAACAGAAAATTTGCTATTTACTAT 712
NbenIDA1B      ACATTTGAAGGTATACAGAAAAGAAAATGCATACAAAACAGAAAATTTGGTATTTACTAT 724
NtabIDA1A      ACATTTGAAGGTATACAGAAAAGAGAATGCATACAAAACAGAAAATTTGCTATTTACTAT 742
NsylIDA1       ACATTTGAAGGTATACAGAAAAGAGAATGCATACAAAACAGAAAATTTGCTATTTACTAT 736
               *********** ************ ************************ **********

NbenIDA1A      GCTACCAAAAGGGGTTCCAATTCCTCCTTCTGCTCCATCTAAGAGN--------------- 773
NtabIDA1B      GCTACCAAAAGGGGTTCCAATTCCTCCTTCTGCTCCATCCAAAAGGCACAATGCTGTTAT 772
NtomIDA1       GCTACCAAAAGGGGTTCCAATTCCTCCTTCTGCTCCATCCAAAAGGCACAATGCTGTTAT 772
NbenIDA1B      GCTACCAAAAGGGGTTCCAATTCCTCCTTCTGCTCCATCTAAGAGACACAATGCTTTTGT 784
NtabIDA1A      GCTACCAAAAGGGGTTCCAATTCCTCCTTCTGCTCCATCTAAGAGACACAATGCTTTTGT 802
NsylIDA1       GCTACCAAAAGGGGTTCCAATTCCTCCTTCTGCTCCATCTAAGAGACACAATGCTTTTGT 796
               *************************************** ** **

NbenIDA1A      ------------------------------------------------------------ 773
NtabIDA1B      GGACTCTTCACCTCAAAATTTCAATATGCTACCAAAAGGTGTTCCTATTCCTCCTTCTGC 832
NtomIDA1       GGACTCTTCACCTCAAAAT----------------------------------------- 791
NbenIDA1B      GGACTCTTCACCTCAAAATTGA-------------------------------------- 806
NtabIDA1A      GGACTCTTCTCCTCAAAAT----------------------------------------- 821
NsylIDA1       GGACTCTTCTCCTCAAAAT----------------------------------------- 815


NbenIDA1A      ----------------------------------------      773
NtabIDA1B      ACCATCCAAAAGGCACAATTATTATGTGAACTCTTATCCT      872
NtomIDA1       ----------------------------------------      791
NbenIDA1B      ----------------------------------------      806
NtabIDA1A      ----------------------------------------      821
NsylIDA1       ----------------------------------------      815
```
