# Supplementary material for: Differential expression of IDA (INFLORESCENCE DEFICIENT IN ABSCISSION)-like genes in Nicotiana benthamiana during corolla abscission, stem growth and water stress
Source: BMC Plant Biol. 2020 Jan 20;20:34. doi: 10.1186/s12870-020-2250-8 (PMC6971993; doi:10.1186/s12870-020-2250-8)
Supplement: Supplementary file 2 — Additional file 2: Solanaceae HSL family.pdf. HAE-like gene families in species of the Solanaceae family, (genome localization from different Sol Genomics Network databases [50]). [file 12870_2020_2250_MOESM2_ESM.pdf]

| Gene name | Sequence ID | Genome localization | Orientation |
|---|---|---|---|
| NsylHAE | Nsyl_KD975002.1 | Nsyl_KD975002.1:55601-58800 | Forward |
| NsylHSL1 | Nsyl_KD937107.1 | Nsyl_KD937107.1:9901-13300 | Forward |
| NsylHSL2 | Nsyl_KD957655.1 | Nsyl_KD957655.1:30001-33700 | Reverse |
| NtomHAE | Ntom_KB968468.1 | Ntom_KB968468.1:8101-11200 | Reverse |
| NtomHSL1 | Ntom_KB958681.1 | Ntom_KB958681.1:11701-15400 | Forward |
| NtomHSL2 | Ntom_KB957417.1 | Ntom_KB957417.1:73401-77200 | Reverse |
| NtabHAE.1 | Ntab-BX_AWOK-SS18352 | Ntab-BX_AWOK-SS18352:54701-58000 | Forward |
| NtabHAE.2 | Ntab-BX_AWOK-SS2766 | Ntab-BX_AWOK-SS2766:217401-220500 | Reverse |
| NtabHSL1.1 | Ntab-BX_AWOK-SS4971 | Ntab-BX_AWOK-SS4971:8301-12000 | Forward |
| NtabHSL1.2 | Ntab-BX_AWOK-SS11846 | Ntab-BX_AWOK-SS11846:218301-222000 | Forward |
| NtabHSL2.1 | Ntab-BX_AWOK-SS5522 | Ntab-BX_AWOK-SS5522:246701-250400 | Forward |
| NtabHSL2.2 | Ntab-BX_AWOK-SS17840 | Ntab-BX_AWOK-SS17840:84701-88400 | Forward |
| NbenHAE.1 | Niben101Scf09774Ctg001 | Niben101Scf09774Ctg001:1101-4300 | Forward |
| NbenHAE.2 | Niben101Scf05190Ctg007 | Niben101Scf05190Ctg007:1-2400 | Forward |
| NbenHSL1.1 | Niben101Scf03169Ctg027 | Niben101Scf03169Ctg027:801-3900 | Forward |
| NbenHSL1.2 | Niben101Scf11552Ctg025 | Niben101Scf11552Ctg025:201-1700 | Reverse |
| NbenHSL2.1 | Niben101Scf08143Ctg022 | Niben101Scf08143Ctg022:18001-21800 | Forward |
| NbenHSL2.2 | Niben101Scf02417Ctg009 | Niben101Scf02417Ctg009:4601-8400 | Forward |
| SolycHAE | Solyc07g053600.2 | SL3.0ch07:62148101-62151900 | Reverse |
| SolycHSL1.1 | Solyc03g006300.1 | SL3.0ch03:900801-904500 | Reverse |
| SolycHSL1.2 | Solyc02g077630.2 | SL3.0ch02:43076801-43080600 | Reverse |
| SolycHSL2 | Solyc02g091860.2 | SL3.0ch02:53739501-53743100 | Forward |
| StubHAE | PGSC0003DMP400016577 | PGSC0003DMS000001958:121901-125200 | Reverse |
| StubHSL1.1 | PGSC0003DMP400022697 | PGSC0003DMS000002817:272401-276100 | Forward |
| StubHSL1.2 | PGSC0003DMP400054963 | PGSC0003DMS000000491:167401-171200 | Forward |
| SmelHAE | Sme2.5_02596.1 | Sme2.5_02596.1:601-4600 | Forward |
| SmelHSL1 | Sme2.5_00787.1_g00015.1 | Sme2.5_00787.1:80801-84500 | Reverse |
| SmelHSL2 | Sme2.5_01937.1_g00002.1 | Sme2.5_01937.1:7701-9900 | Forward |
| CaHAE | CA00g84190 | CA00g84190:101-983 | Forward |
| CaHSL1 | CA02g15510 | CA02g15510:101-1002 | Reverse |
| CaHSL2 | CA02g24590 | CA02g24590:101-984 | Forward |
