# Supplementary figures and images for: Differential expression of IDA (INFLORESCENCE DEFICIENT IN ABSCISSION)-like genes in Nicotiana benthamiana during corolla abscission, stem growth and water stress
Source: BMC Plant Biol. 2020 Jan 20;20:34. doi: 10.1186/s12870-020-2250-8 (PMC6971993; doi:10.1186/s12870-020-2250-8)

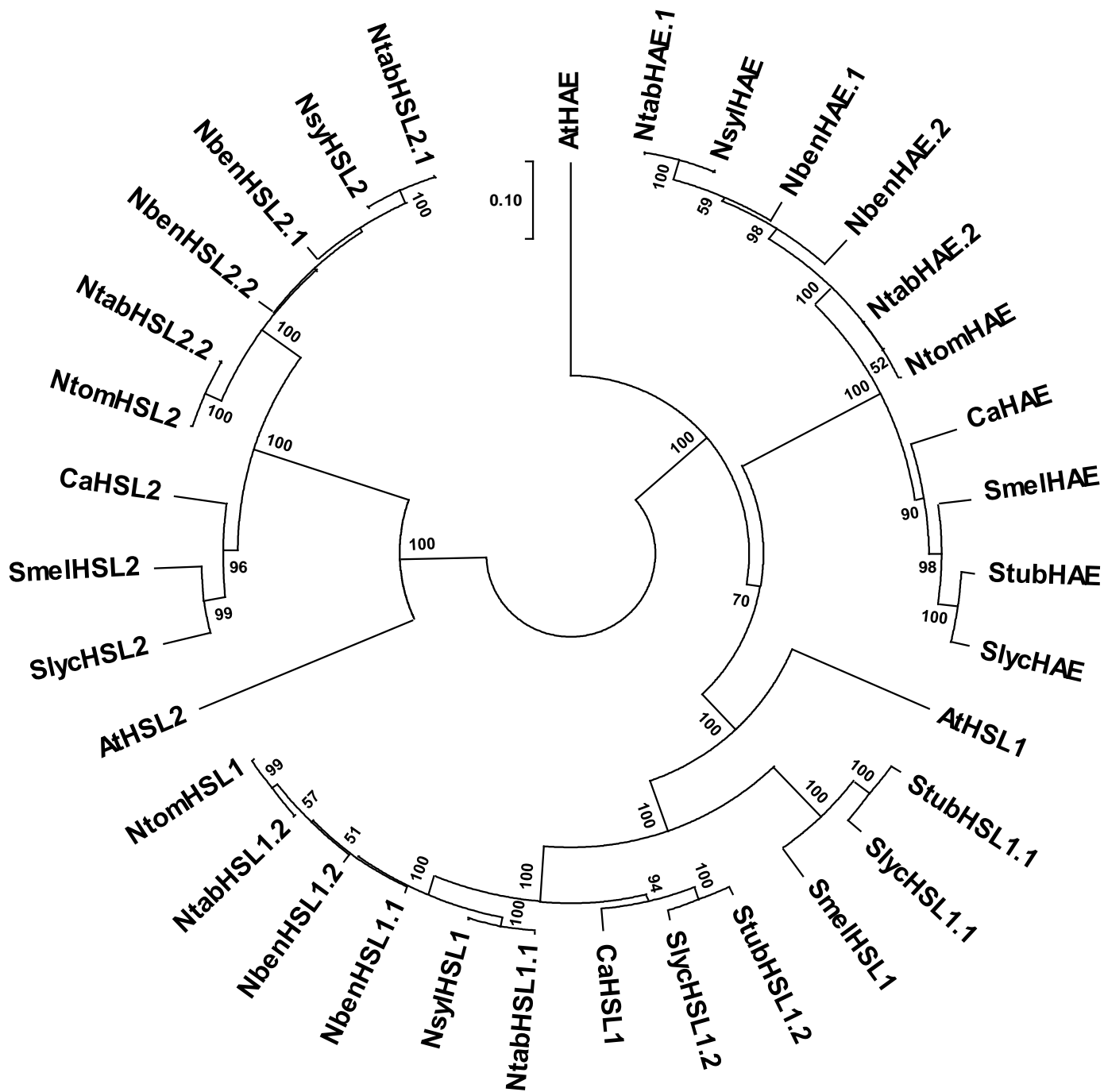

Supplement: Supplementary file 3 — Additional file 3: HAE-like peptides phylogenetic tree.pdf. Circular phylogenetic tree of HAE-like peptides of Arabidopsis thaliana and several species of the Solanaceae family (N. benthamiana, N. tabacum, N. sylvestris, N. tomentosiformis, S. lycopersicum, S. tuberosum, S. melongena and C. annuum). [file 12870_2020_2250_MOESM3_ESM.pdf]
