# Supplementary material for: Differential expression of IDA (INFLORESCENCE DEFICIENT IN ABSCISSION)-like genes in Nicotiana benthamiana during corolla abscission, stem growth and water stress
Source: BMC Plant Biol. 2020 Jan 20;20:34. doi: 10.1186/s12870-020-2250-8 (PMC6971993; doi:10.1186/s12870-020-2250-8)
Supplement: Supplementary file 6 — Additional file 6: qPCR primers.pdf. Primers used for quantitative PCR analysis. [file 12870_2020_2250_MOESM6_ESM.pdf]

Primer name	Sequence
NbenPP2A_F	GACCCTGATGTTGATGTTCGCT
NbenPP2A_R	GAGGGATTTGAAGAGAGATTTC
NbenIDA1A_F	TGAAGCAAGACCAGGAAGAATG
NbenIDA1A_R	GGAACCCCTTTTGGTAGCATAG
NbenIDA1B_F	CCAAAAGGGGTTCCAATTCCTC
NbenIDA1B_R	GAGGTGAAGAGTCCACAAAAGC
NbenIDA2A_F	ATCAATGGTGGCAACGACGA
NbenIDA2A_R	TTGAGCCAATCACCTTTCAAATATTCAT
NbenIDA2B_F	GATCAATGGTGACAACAACAG
NbenIDA2B_R	TGAACCAATTTTCTTTCAGATTCTTTT
NbenIDA3A_F	TCTTGGCTGATTATCACCATGC
NbenIDA3A_R	AAGGAGCAGAAGGTGGAATAGG
NbenIDA3B_F	TGTTAAGCCATTGCCTAATTCCC
NbenIDA3B_R	CCATTGTGCCTTTTCGAAGGAG
NbenIDA4_F	GGCAACGCACACACAATTTC
NbenIDA4_R	TGATACCATTGTGCCGTTGG
NbenIDA5A_F	CCCAAGTAGCCAAAGAAACTCTC
NbenIDA5A_R	TTTCTTGATGGACCAGAAGCTG
NbenIDA5B_F	GTCATTGTCATGGTTCAAGAAGC
NbenIDA5B_R	GGCAACAAGTTCCAAAAATGGC
NbenHAE.1_F	AATTGGGACTTTGCCAGTGC
NbenHAE.1_R	TGCAAGCTGAGTGGAATTGC
NbenHAE.2_F	TTGCTGAACAGTTGCAAGGC
NbenHAE.2_R	ACATCGCGGTGTACAATTGG
NbenHSL2.1_F	TATAGCTTTGGCGTGGTCCTG
NbenHSL2.1_R	CGACTAGCCGGTTCAAATCAAG
NbenHSL2.2_F	ACTGCATTTCAACGGGTTGG
NbenHSL2.2_R	TGCCCGCTTTTCAGTTTGAC
