# Supplementary material for: Differential expression of IDA (INFLORESCENCE DEFICIENT IN ABSCISSION)-like genes in Nicotiana benthamiana during corolla abscission, stem growth and water stress
Source: BMC Plant Biol. 2020 Jan 20;20:34. doi: 10.1186/s12870-020-2250-8 (PMC6971993; doi:10.1186/s12870-020-2250-8)
Supplement: Supplementary file 4 — Additional file 4: Alignment IDA-like prepropeptides.pdf. Sequence alignment of IDA-like peptides from several species of the Solanaceae family (N. benthamiana, N. tabacum, N. sylvestris, N. tomentosiformis, S. lycopersicum, S. tuberosum, S. melongena and C. annuum) and from Arabidopsis. The CINEMA color scheme is used to inform about the chemical nature of the amino acid residues in the EPIP domain (blue, polar positive; red, negative; green, neutral; white, non-polar aliphatic; purple, ocher and yellow, aromatic residues). [file 12870_2020_2250_MOESM4_ESM.pdf]

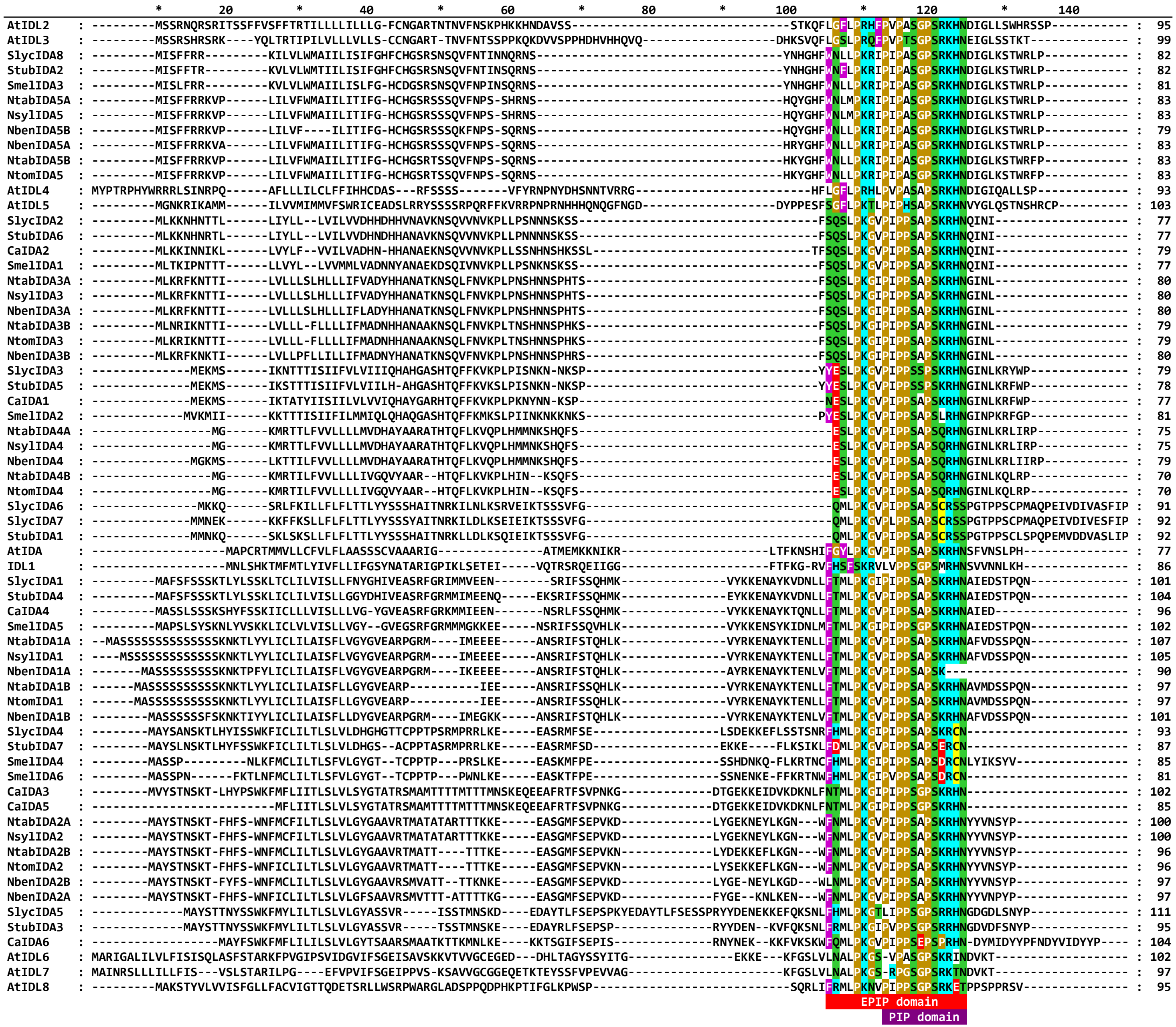
* 20 * 40 * 60 * 80 * 100 * 120 * 140
AtIDL2 : 95
AtIDL3 : 99
SlycIDA8 : 82
StubIDA2 : 82
SmelIDA3 : 81
NtabIDA5A : 83
NsylIDA5 : 83
NbenIDA5B : 79
NbenIDA5A : 83
NtabIDA5B : 83
NtomIDA5 : 83
AtIDL4 : 93
AtIDL5 : 103
SlycIDA2 : 77
StubIDA6 : 77
CaIDA2 : 79
SmelIDA1 : 77
NtabIDA3A : 80
NsylIDA3 : 80
NbenIDA3A : 80
NtabIDA3B : 79
NtomIDA3 : 79
NbenIDA3B : 80
SlycIDA3 : 79
StubIDA5 : 78
CaIDA1 : 77
SmelIDA2 : 81
NtabIDA4A : 75
NsylIDA4 : 75
NbenIDA4 : 79
NtabIDA4B : 70
NtomIDA4 : 70
SlycIDA6 : 91
SlycIDA7 : 92
StubIDA1 : 92
AtIDA : 77
IDL1 : 86
SlycIDA1 : 101
StubIDA4 : 104
CaIDA4 : 96
SmelIDA5 : 102
NtabIDA1A : 107
NsylIDA1 : 105
NbenIDA1A : 90
NtabIDA1B : 97
NtomIDA1 : 97
NbenIDA1B : 101
SlycIDA4 : 93
StubIDA7 : 87
SmelIDA4 : 85
SmelIDA6 : 81
CaIDA3 : 102
CaIDA5 : 85
NtabIDA2A : 100
NsylIDA2 : 100
NtabIDA2B : 96
NtomIDA2 : 96
NbenIDA2B : 97
NbenIDA2A : 97
SlycIDA5 : 111
StubIDA3 : 95
CaIDA6 : 104
AtIDL6 : 102
AtIDL7 : 97
AtIDL8 : 95
EPIP domain
PIP domain
